# Culture Condition Optimization and Pilot Scale Production of the M12 Metalloprotease Myroilysin Produced by the Deep-Sea Bacterium *Myroides profundi* D25

**DOI:** 10.3390/molecules200711891

**Published:** 2015-06-29

**Authors:** Xuan Shao, Li-Yuan Ran, Chang Liu, Xiu-Lan Chen, Xi-Ying Zhang, Qi-Long Qin, Bai-Cheng Zhou, Yu-Zhong Zhang

**Affiliations:** 1 State Key Laboratory of Microbial Technology, Shandong University, Jinan 250100, China; E-Mails: swjd2010@163.com (X.S.); ranly1020@126.com (L.-Y.R.); wumingyoucai@aliyun.com (C.L.); cxl0423@sdu.edu.cn (X.-L.C.); zhangxiying@sdu.edu.cn (X.-Y.Z.); zhangyz@sdu.edu.cn (Y.-Z.Z.); 2 Marine Biotechnology Research Center, Shandong University, Jinan 250100, China; E-Mail: bczhou@qdio.ac.cn

**Keywords:** elastase, myroilysin, single factor experiments, small scale fermentation, pilot scale fermentation, protease, marine sediment

## Abstract

The protease myroilysin is the most abundant protease secreted by marine sedimental bacterium *Myroides profundi* D25. As a novel elastase of the M12 family, myroilysin has high elastin-degrading activity and strong collagen-swelling ability, suggesting its promising biotechnological potential. Because myroilysin cannot be maturely expressed in *Escherichia coli*, it is important to be able to improve the production of myroilysin in the wild strain D25. We optimized the culture conditions of strain D25 for protease production by using single factor experiments. Under the optimized conditions, the protease activity of strain D25 reached 1137 ± 53.29 U/mL, *i.e.*, 174% of that before optimization (652 ± 23.78 U/mL). We then conducted small scale fermentations of D25 in a 7.5 L fermentor. The protease activity of strain D25 in small scale fermentations reached 1546.4 ± 82.65 U/mL after parameter optimization. Based on the small scale fermentation results, we further conducted pilot scale fermentations of D25 in a 200 L fermentor, in which the protease production of D25 reached approximately 1100 U/mL. These results indicate that we successfully set up the small and pilot scale fermentation processes of strain D25 for myroilysin production, which should be helpful for the industrial production of myroilysin and the development of its biotechnological potential.

## 1. Introduction

Particulate organic nitrogen is abundant in marine sediments, and bacterial enzymatic activity in sediments is generally the initial and rate-limiting step in nitrogen recycling [1]. It has been reported that a large number of protease-producing bacteria exist in marine sediments that secrete various proteases to decompose the proteins in sedimentary organic nitrogen. For example, Olivera *et al*. screened 19 protease-producing strains from sub-Antarctic sediments [2]; Zhou *et al*. screened 78 protease-producing strains from the sediments of the South China Sea and 105 protease-producing strains from the Coastal Sediments of King George Island, Antarctica, and they also found that these strains secrete various serine proteases and metalloproteases [3,4]. herefore, marine sediments are good resources for exploiting novel proteases with biotechnological potential.

In recent years, many proteases from bacteria isolated from marine sediments have been studied. Three extracellular proteases, MCP-01, MCP-02 and MCP-03, from *Pseudoalteromonas* sp. SM9913 isolated from deep sea sediment have been studied. MCP-01 is a novel serine collagenolytic protease of the S8 family [5,6,7], MCP-02 is a metalloprotease of the M4 family [5,8], and MCP-03 is a cold-adapted and salt-tolerant protease of the S8 family [9]. A cold-active alkaline protease from *Pseudomonas* sp. strain DY-A that was isolated from a 5255 m deep sediment of the East Paciﬁc is also characterized [10]. The protease, pseudoalterin, from a South China sediment strain *Pseudoalteromonas* sp. CF6-2 is a novel elastase of the M23 family [3,11]. These studies indicate that the bacterial proteases from marine sediments usually have some novel characteristics, and may have promosing potential in various industries, such as detergents, food processing and pharmaceutical engineering. Fernandes has documented the potentials of marine proteases in the food industry [12].

*Myroides profundi* D25 is a protease-producing bacterium isolated from a 1245 m water depth sediment near southern Okinawa [13]. Myroilysin is the most abundant protease secreted by D25, and it is a novel metalloprotease belonging to the M12 family. Myroilysin has high elastinolytic activity, and is the first elastase reported in the M12 family [14]. Besides elastinolytic activity, myroilysin also has strong collagen-swelling ability, but with little collagenolytic activity [14]. These characteristics of myroilysin suggest that this protease may act as a good collagen modifier, and therefore can be used in leather softening. Collagen has a wide range of uses in the biomedical field, for an example, as a material for tissue engineering and hemostatic sponge [15,16,17,18]. Therefore, myroilysin may have promising potentials in these biomedical fields. In addition, as an efficient elastase, myroilysin may have potentials to be used in meat tenderization in the food processing industry.

Due to its promising biotechnological potential, it is necessary to improve the production of myroilysin for its application. Heterogenous expression, such as in *Escherichia coli*, is now the common way to improve enzyme production [19,20]. However, because the M12 proteases are not autoprocessed during maturation [21], it is almost impossible to express them into a mature form in a heterogenous expression system, thus, it is important to improve the production of these enzymes in their wild strains by optimizing the fermentation conditions. We have tried to express myroilysin in *E. coli*, but failed to obtain an active recombinant enzyme, most likely because myroilysin is also a non-autoprocessed M12 protease. We previously developed a cheap fermentation medium for the production of myroilysin in strain D25 [14]. With this medium as a basal fermentation medium, in this study, we optimized the culture conditions of D25 by using single factor experiments to improve the production of myroilysin of strain D25. Based on the optimized culture conditions, we further conducted small scale fermentations in a 7.5 L fermentor and the pilot scale fermentations in a 200 L fermentor. The results lay a good foundation for developing the biotechnological potential of myroilysin.

## 2. Results and Discussion

### 2.1. Effects of Culture Temperature and Inoculation Amount on the Production of Myroilysin

The fermentation medium we previously developed for the production of myroilysin in strain D25 contained (*w*/*v*) 2% corn powder, 2% bean powder, 1% wheat bran, 0.4% Na_2_HPO_4_, 0.03% KH_2_PO_4_, 0.1% CaCl_2_, and artificial seawater (pH 8.0) [14]. This medium is called the basal medium in this study. Based on the basal medium, we optimized the culture conditions of D25 for protease production by single factor experiments.

**Figure 1:**
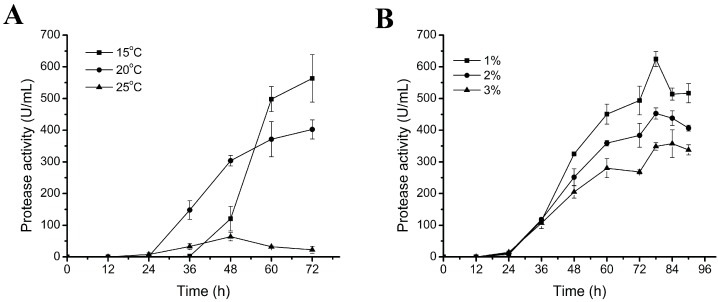
Effects of culture temperature (**A**) and inoculation amount (**B**) on the protease production of D25 in the basal medium. The graph shows data from triplicate treatments (mean ± S.D.).

We first investigated the effect of culture temperature on the production of myroilysin in the basal medium. As shown in Figure 1A, when D25 was cultured for 72 h at 15 °C, 20 °C and 25 °C, respectively, the broth at 15 °C showed the highest protease activity, indicating that D25 had the highest myroilysin production when cultured at 15 °C. This is accordant to the cold-adapted characteristic of D25, which was isolated from permanently cold marine sediment [13]. We then investigated the effect of inoculation amount on the production of myroilysin. As shown in Figure 1B, when cultured at 15 °C, the broth with 1% inoculation amount showed the highest protease activity, and the broth with the inoculation amount more than 1% showed lower protease activity. Therefore, 1% inoculation amount was used in the following experiments.

### 2.2. Optimization of the Contents of Carbon Source and Nitrogen Source in the Medium

**Figure 2:**
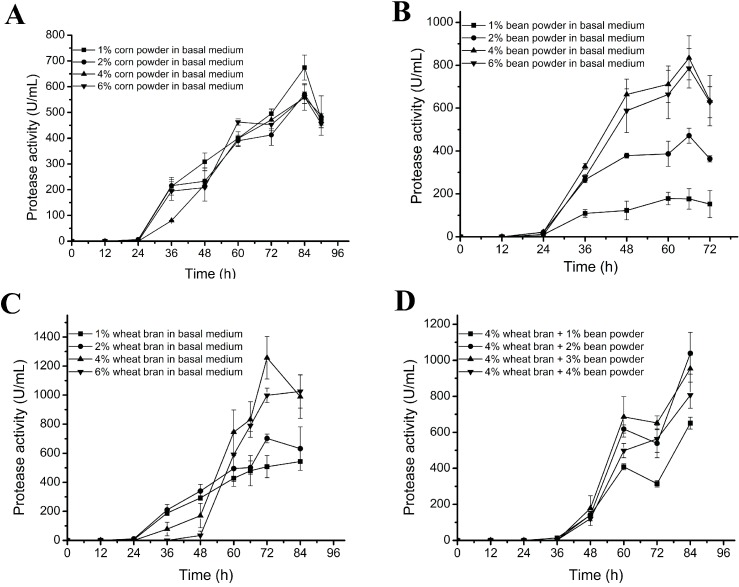
Effects of the contents of carbon source and nitrogen source in the medium on the protease production of D25. (**A**) Effect of corn powder content with 2% bean powder and 1% wheat bran in the medium; (**B**) Effect of bean powder content with 2% corn powder and 1% wheat bran in the medium; (**C**) Effect of wheat bran content with 2% corn powder and 2% bean powder in the medium; (**D**) Effect of bean powder content with 2% corn powder and 4% wheat bran in the medium. For (**A**–**C**), D25 was cultured at 15 °C and 200 rpm with a tested single factor being changed in the basal medium. For (**D**), D25 was cultured at 15 °C and 200 rpm with bean powder content being changed in a revised basal medium containing 4% wheat bran instead of 2% wheat bran. The graph shows data from triplicate treatments (mean ± S.D.).

The basal medium contained 2% corn powder as carbon source, and 2% bean powder and 1% wheat bran as nitrogen source [14]. We determined the optimal content of each of these components in the medium for myroilysin production. The protease production of D25 showed only slight differences in the media containing 1%–6% corn powder (Figure 2A), indicating that the content of corn powder had little effect on the protease production of D25.

In contrast, the contents of both bean powder and wheat bran in the medium had significant effects on the protease production of D25. When bean powder was set as a single factor variable in the basal medium, the protease production reached the highest (921 ± 150 U/mL) in the medium containing 4% bean powder, increasing by 35% compared to that in the basal medium (Figure 2B). The content of wheat bran in the medium showed an even more significant effect on the protease production of D25 than bean powder. With wheat bran as a single factor variable in the basal medium, the protease production peaked in the medium containing 4% wheat bran, which was approximately two-fold (1256 ± 146 U/mL) of that in the basal medium (Figure 2C). However, when the medium contained 4% bean powder and 4% wheat bran, the protease production was decreased to 806 ± 72 U/mL, probably because the medium was too thick. Thus, we further investigated the optimal content of bean powder in the medium containing 4% wheat bran. As shown in Figure 2D, in the medium containing 4% wheat bran, the protease production of D25 reached the highest when the content of bean powder was 2%, indicating that 2% bean powder is optimal in the presence of 4% wheat bran in the medium. We noticed that there was a small difference in the maximal protease activity between the same treatments in Figure 2C,D, which should result from the difference in different batches. Taken together, these results indicate that the content of nitrogen source in the medium has significant effect on the protease production of D25, and that the medium containing 1% corn powder, 2% bean powder and 4% wheat bran is optimal for the protease production of D25.

### 2.3. Protease Production of D25 under the Optimal Conditions

Based on the results of the above single factor experiments, the improved conditions for the protease production of D25 can be concluded as: the medium contains (*w*/*v*) 1% corn powder, 4% wheat bran, 2% bean powder, 0.4% Na_2_HPO_4_, 0.03% KH_2_PO_4_, 0.1% CaCl_2_, and artificial seawater, with the initial pH of 8.0; the inoculation amount is 1%; the culture temperature is 15 °C and the stirring speed is 200 rpm.

**Figure 3:**
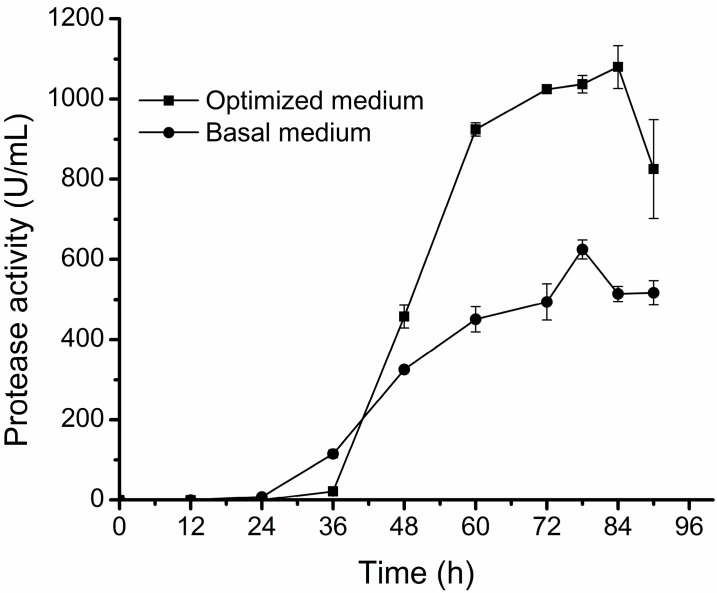
Comparison of the protease production of D25 in the optimized medium and in the basal medium. D25 was cultured at 15 °C and 200 rpm with 1% inoculation amount. The graph shows data from triplicate treatments (mean ± S.D.).

We then conducted D25 fermentation in a 500 mL flask with 50 mL of the optimized medium or of the basal medium under the optimal culture conditions. The result showed that the protease activity in the optimized medium reached 1137 ± 53.29 U/mL, much higher than that in the basal medium (652 ± 23.78 U/mL) (Figure 3). This result shows that the protease production of D25 is significantly improved by optimizing culture conditions.

### 2.4. Small Scale Fermentation of D25

**Figure 4:**
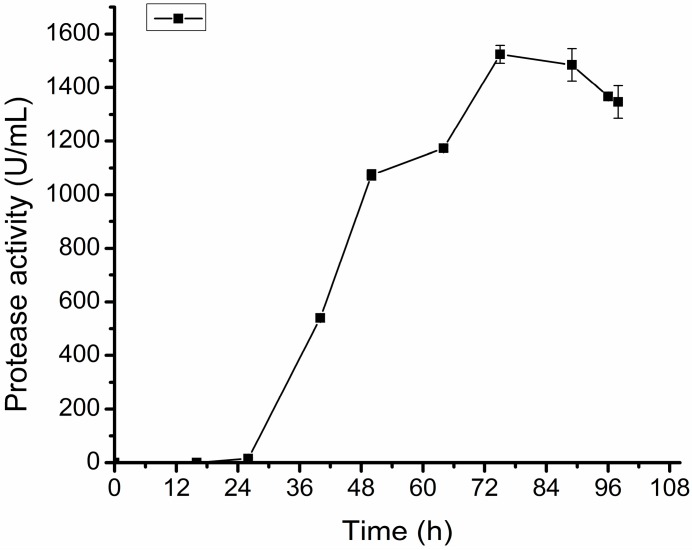
The protease production of D25 in a 7.5 L fermentor under the determined small scale fermentation process. The graph shows data from triplicate treatments (mean ± S.D.).

As an efficient elastase with high collagen-swelling ability, myroilysin has promising biotechnological potentials, which promote us to conduct the small and pilot scale fermentation of D25 to lay a foundation for the industrial production of myroilysin. Using the optimized medium, we first conducted small scale fermentations in a 7.5 L fermentor. After six fermentation batches, we optimized the parameters for D25 fermentation in a 7.5 L fermentor, and set up a small scale fermentation process. Five liters of medium was put into the 7.5 L fermentor and sterilized, and 1% volume of D25 seed culture was inoculated into the medium, which was then cultured at 15 °C with a stirring speed of 300 rpm, and an aeration rate of 0.4 L/min. After 24 h culturation, when the strain growth entered into the logarithmic growth phase, the stirring speed was adjusted to 450 rpm, and the aeration rate was adjusted to 0.8 L/min to increase the dissolved oxygen in the culture to meet the increase of oxygen consumption. After 48 h culture, the strain growth entered into the late logarithmic growth phase, and oxygen consumption in the culture was reduced due to the decrease of the growth rate of the strain. To reduce the damage of the shear force on the protease, the stirring speed was decreased to 400 rpm, which was maintained to the end of fermentation. By using this fermentation technique, the protease production of D25 reached 1546.4 ± 82.65 U/mL (Figure 4), which was 36% higher than that of the flask fermentation.

### 2.5. Pilot Scale Fermentation of D25

**Figure 5:**
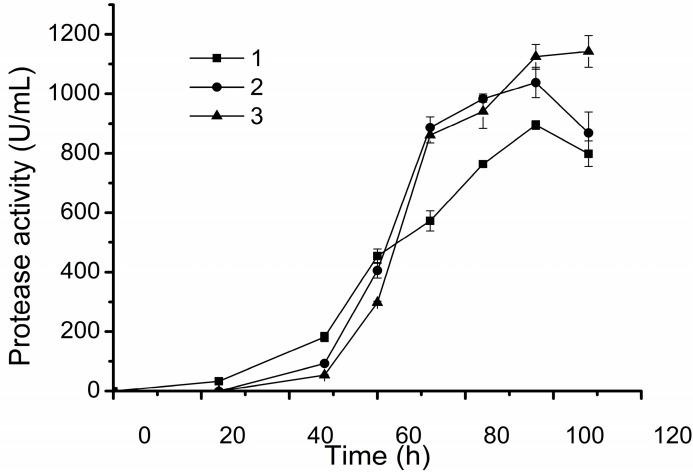
The protease activity of the D25 broth of the pilot scale fermentation under different conditions. Line 1, the protease activity of the fermentation broth with the aeration rate of 1 m^3^/h and the stirring speed of 90 rpm. Line 2, the protease activity of the fermentation broth with the aeration rate of 2 m^3^/h and the stirring speed of 80 rpm. Line 3, the protease activity of the fermentation broth with the aeration rate of 2 m^3^/h and the stirring speed of 90 rpm. The graph shows data from triplicate treatments (mean ± S.D.).

Based on the results of the single factor experiments and the small scale fermentations, we further conducted pilot scale fermentations of D25 in a 200 L fermentor. As in the small scale fermentations, the stirring speed and the aeration rate were found to be two important factors for the protease production of D25 in the pilot scale fermentations. As shown in Figure 5, when the aeration rate was 1 m^3^/h, and the stirring speed was 90 rpm, the protease activity of the broth reached 905 ± 14.85 U/mL, which reached 1037 ± 51.24 U/mL under the aeration rate of 2 m^3^/h and the stirring speed of 80 rpm, and 1173 ± 83.92 U/mL under the aeration rate of 2 m^3^/h and the stirring speed of 90 rpm. After optimization, we determined the pilot scale fermentation process of D25 in a 200 L fermentor. The medium, the inoculation amount and the culture temperature are the same as those in the small scale fermentation. The initial aeration rate is 2.4 m^3^/h, which is decreased to 2 m^3^/h after 48 h. The stirring speed is kept at 90 rpm in the whole fermentation process. With this process, the protease activity of the fermentation broth of D25 reached approximately 1100 U/mL.

Although many proteases from marine sedimental bacteria have been characterized, reports on the pilot scale fermentation and industrial production of these proteases are rather less. Zhou *et al.* developed a cheap medium for the production of pseudoalterin by strain *Pseudoalteromonas* sp. CF6-2 with bovine artery powder as an inducer instead of expensive purified elastin. They optimized the fermentation condition for pseudoalterin production by using response surface methodology, which resulted in two fold increase in the pseudoalterin production [22]. The protease myroilysin, secreted by *Myroides profundi* D25, is a novel elastase with strong collagen-swelling ability [14], which may have promising biotechnological potentials. In this study, we optimized the culture conditions of D25 for myroilysin production, and successfully conducted small and pilot scale fermentation processes. The results showed that the myroilysin production of D25 was significantly improved.

## 3. Experimental Section

### 3.1. Strain and Media

*Myroides profundi* D25 was previously isolated from a 1245 m deep-sea water depth sediment at 24°47.19′N, 122°29.30′E near southern Okinawa [5]. The strain D25 was cryopreserved at −80 °C supplemented with 15% glycerol. At the same time, it was routinely cultured at 15 °C for 2 days on a plate containing 10 g/L peptone, 5 g/L yeast extract, artificial sea water and 15 g/L agar (pH 8.0), which was then stored at 4 °C for short term use. The basal fermentation medium for myroilysin production of D25 contained (*w*/*v*) 2% corn powder, 1% wheat bran, 2% bean powder, 0.4% Na_2_HPO_4_, 0.03% KH_2_PO_4_, 0.1% CaCl_2_, with an initial pH of 8.0. The optimized fermentation medium contained (*w*/*v*) 1% corn powder, 4% wheat bran, 2% bean powder, 0.4% Na_2_HPO_4_, 0.03% KH_2_PO_4_, 0.1% CaCl_2_, pH 8.0. All chemicals used in this study were of analytical reagent grade. Wheat bran, bean powder and corn powder were purchased from a local farmer market.

### 3.2. Inoculum Preparation and Flask Fermentation

For inoculum preparation, D25 was inoculated into a marine broth medium composed of 10 g/L peptone, 5 g/L yeast extract and artificial sea water (pH 8.0), and incubated at 20 °C, 200 rpm for 24 h. The culture was used as inoculum for D25 fermentation for protease production. For fermentation, 1% (*v*/*v*) inoculum with an OD_600_ of approximately 1.5 was inoculated into 50 mL medium in a 500 mL Erlenmeyer flask, which was then incubated at 15 °C, 200 rpm.

### 3.3. Assay of the Protease Activity

The protease activity was assayed with casein as substrate as previously described [5]. Briefly, the fermentation culture of D25 was centrifuged at 13,000 rpm, 4 °C for 10 min, and then the protease activity of the supernatant was measured after appropriately diluted with 50 mM Tris-HCl (pH 9.5). A mixture of 0.1 mL 2% (*w*/*v*) casein solution and 0.1 mL diluted supernatant was incubated at 50 °C for 10 min. Then the reaction was stopped by 0.2 mL 0.4 M trichloroacetic acid. In the control sample, the mixture of 0.1 mL diluted supernatant and 0.2 mL 0.4 M trichloroacetic acid was incubated at 50 °C for 10 min, and then 0.1 mL 2% (*w*/*v*) casein solution was added. The samples were centrifuged at 13,000 rpm for 5 min. After centrifugation, 0.1 mL supernatant was mixed with 0.5 mL 0.4 M Na_2_CO_3_, and 0.1 mL Folin & Ciocalteu’s phenol reagent. The mixture was incubated at 40 °C for 20 min, and then the absorption at 660 nm of the mixture was measured. One unit of enzyme activity is defined as the amount of enzyme that catalyzes the hydrolysis of casein to generate 1 μmol tyrosine per min.

### 3.4. Optimization by Single Factor Experiments

Five single factors were detected for their influences on the production of myroliysin, including the content of wheat bran (1%, 2%, 4% or 6%), the content of bean powder (1%, 2%, 4% or 6%), the content of corn powder (1%, 2%, 4% or 6%), culture temperature (15, 20 or 25 °C) and the inoculation amount (1%, 2% or 3%). D25 was cultured under the flask fermentation conditions described above with a tested single factor being changed. All treatments were performed in triplicate.

### 3.5. Small Scale Fermentation and Pilot Scale Fermentation

For small scale fermentation, 1% (*v*/*v*) inoculum was inoculated into 5 L the improved fermentation medium in a 7.5 L fermenter (Bioflo & Celligen 310 fermentor, New Brunswick Scientific, Edison, NJ, USA). For pilot scale fermentation, 1% (*v*/*v*) inoculum was inoculated into 140 L the optimized fermentation medium in a 200 L fermenter (GHJ9-100 fermenter, Shanghai Guoqiang Biochemical Engineering Equipment Co., Ltd., Shanghai, China). The fermentation temperature was 15 °C. The stirring speed and the aeration rate were optimized according to the protease production.

## 4. Conclusions

Optimizing culture conditions is important for improving bacterial enzyme production, and setting up small and pilot fermentation processes provides an important basis for the industrial production of bacterial enzymes. Myroilysin, the most abundant protease secreted by *Myroides profundi* D25, is a novel elastase with promising biotechnological potential. In this study, we optimized the culture conditions of D25 for myroilysin production using single factor experiments. Under the optimized conditions, the protease production of D25 reached 1137 ± 53.29 U/mL, 174% of that before optimization, showing that our culture condition optimization brings a significant improvement of the myroilysin production. Based on the optimized conditions of the flask fermentation, we further determined the small scale and pilot scale fermentation processes of strain D25 for myroilysin production in succession. The protease production reached 1500 U/mL in the small scale fermentation iand 1100 U/mL in the pilot scale fermentation. These results indicate that we successfully set up the pilot scale fermentation process for the myroilysin production of strain D25, which lay a good foundation for the industrial production of myroilysin and the development of its biotechnological applications.
